# The Potential of Polymers and Glass to Enhance Hydrogen Storage Capacity: A Mathematical Approach

**DOI:** 10.3390/ma17246065

**Published:** 2024-12-12

**Authors:** Andrei Ratoi, Corneliu Munteanu, Dan Eliezer

**Affiliations:** 1 Mechanical Engineering, Mechatronics and Robotics Department, Mechanical Engineering Faculty, “Gheorghe Asachi” Technical University of Iasi, 700050 Iasi, Romania; andrey_ratoi@yahoo.com (A.R.); cornelmun@gmail.com (C.M.); 2 Technical Sciences Academy of Romania, 26 Dacia Boulevard, 030167 Bucharest, Romania; 3 Department of Material Engineering, Ben-Gurion University of the Negev, Beer-Sheva 8410501, Israel

**Keywords:** hydrogen storage, gravimetric capacity, volumetric capacity, polymers, composite materials, capillary arrays

## Abstract

This manuscript contributes to understanding the role of hydrogen in different materials, emphasizing polymers and composite materials, to increase hydrogen storage capacity in those materials. Hydrogen storage is critical in advancing and optimizing sustainable energy solutions that are essential for improving their performance. Capillary arrays, which offer increased surface area and optimized storage geometries, present a promising avenue for enhancing hydrogen uptake. This work evaluates various polymers and glass for their mechanical properties and strength with 700 bar inner pressure loads within capillary tubes. A theoretical mathematical approach was employed to quantify the impact of material properties on storage capacity. Our results demonstrate that certain polymers (e.g., Zylon AS, Dyneema SK99) and glass types (S-2 Glass) exhibit superior hydrogen storage potential due to their enhanced strength and low density. These findings suggest that integrating the proposed materials into capillary array systems can significantly improve hydrogen storage efficiency (15–37 wt.% and 37–40 g/L), making them viable candidates for next-generation energy storage systems. This study provides valuable insights into material selection and structural design strategies for high-capacity hydrogen storage technologies.

## 1. Introduction

The demand for efficient and sustainable energy storage solutions is at the forefront of scientific and technological innovation, particularly in the context of hydrogen, which is considered a clean and renewable energy carrier [1]. Hydrogen storage is a critical challenge in advancing hydrogen-based energy systems, as its low density and small molecular size present difficulties in developing materials that can store significant quantities of the gas efficiently [2]. Achieving high gravimetric and volumetric storage capacities is vital for future hydrogen technologies, especially in transportation and grid energy storage applications. The U.S. Department of Energy (DOE) has set ambitious targets for hydrogen storage systems, requiring the development of materials and designs that meet stringent performance metrics in weight and volume [3].

Among the various existing methods explored for hydrogen storage [4,5,6], capillary arrays composed of high-surface-area microtubes have emerged as a promising option due to their potential for enhanced hydrogen adsorption and retention [7]. The geometric properties of capillary arrays, including their diameter and arrangement, can be optimized to maximize hydrogen uptake. However, choosing materials for the capillary tubes remains a key challenge. Traditionally, metals have been extensively studied for hydrogen storage [8,9], but often face limitations in terms of gravimetric capacity and hydrogen permeability [10].

In recent years, polymers and glass materials have attracted increasing attention due to their lightweight nature, high mechanical strength, and ability to be engineered with tunable properties [11]. Polymers such as polyetheretherketone (PEEK) and ultrahigh molecular weight polyethylene (UHMWPE) have demonstrated considerable potential for hydrogen storage in high-pressure environments due to their strength-to-weight ratio, durability under extreme conditions, and gas barrier properties [12,13]. Glass, particularly thin-walled tubes, is a possible alternative due to its chemical stability and capacity to withstand extreme pressures without significant deformation [14]. With optimized geometrical designs of capillary arrays, these materials present an exciting frontier for advancing hydrogen storage technologies.

Despite these promising properties, there is an ongoing debate over the optimal material for hydrogen storage systems. While some studies suggest that polymers alone may not provide sufficient gas retention [15], others advocate for hybrid polymer-glass systems [16]. This controversy highlights the need for further research to identify the optimal material and design combinations to meet the DOE’s hydrogen storage targets.

This study evaluates the potential of polymers and glass materials to improve the gravimetric and volumetric capacities of hydrogen storage systems through a combination of material selection and capillary array design. Using a theoretical approach, we assess the mechanical properties of these materials under 700 bar pressure. We then employ a mathematical model to quantify their impact on hydrogen storage performance. We focus on capillary arrays, where the material selection is critical for optimizing the storage capacity and ensuring mechanical stability. Our findings suggest that certain polymer and glass materials can significantly improve gravimetric storage goals when used in capillary array solutions. This study provides valuable insights into the material selection and structural design strategies critical for developing next-generation hydrogen storage systems.

## 2. Materials and Methods

### 2.1. Hydrogen Storage Technology Description

Developing efficient hydrogen storage systems is crucial for enabling the widespread use of hydrogen as an energy carrier. The current major types of hydrogen storage technologies are broadly classified into three categories: compressed gas storage, liquid hydrogen storage, and solid-state storage. Each category has distinct advantages and challenges, with their applications depending on factors such as storage capacity, safety, cost, and system complexity [4,5,6].

Among these, compressed gas storage in high-pressure tanks is the most widely used method for hydrogen storage, particularly for mobile and portable applications. High-pressure tanks are classified into four main types based on the materials used for the liner and reinforcing layers [17]:

- Type I: These tanks are composed entirely of metal, typically steel or aluminum. They are durable but heavy, limiting their gravimetric efficiency.
- Type II: These tanks have a metal liner with partial composite reinforcement, providing a balance between weight and cost but with limited improvement in storage capacity.
- Type III: A metal liner (often aluminum) fully wrapped with a composite material, these tanks offer improved weight reduction and strength, though they are more expensive.
- Type IV: Tanks that use a plastic (polymer) liner with full composite wrapping, making them the lightest option and superior in gravimetric efficiency. However, they are the costliest and require careful design to ensure the control of hydrogen permeability.

Despite the widespread use of high-pressure tanks, they face challenges such as limited volumetric capacity, as can be seen in Table 1 in comparison to DOE’s Targets, high costs, weight, and safety concerns (particularly under extreme operating conditions). As a result, alternative solutions are being explored to enhance hydrogen storage performance.

**Figure 1:**
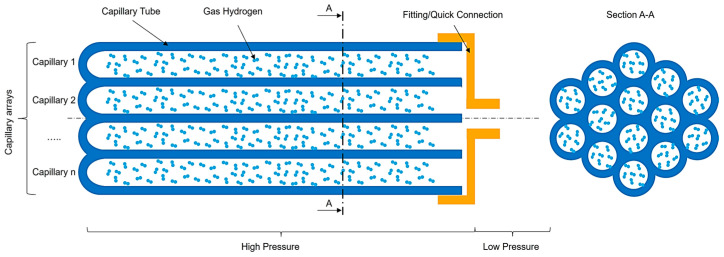
Generic representation of capillary array hydrogen storage in longitudinal and section cut.

Capillary array systems, proposed by C. En [14,19], are an alternative to common type I-IV tanks. These systems offer substantial advantages over traditional high-pressure tanks, particularly in their ability to maximize the volume available for hydrogen storage. Capillary arrays, generically represented in Figure 1, consist of numerous small-diameter tubes arranged in specific geometric configurations, optimizing both gravimetric and volumetric capacities. By leveraging this design, capillary arrays effectively increase the space available for hydrogen, achieving higher efficiency than standard tanks.

One key advantage of capillary arrays is their ability to distribute stress more evenly across the storage system. The close packing of capillaries allows for thinner walls, as the array structure inherently supports better stress distribution. This reduces the storage system’s weight and increases the free volume available for hydrogen, making the storage significantly more efficient. By contrast, traditional type I to IV tanks require thicker walls to handle internal pressure uniformly, which adds to the system’s overall weight and reduces the available internal volume.

**Figure 2:**
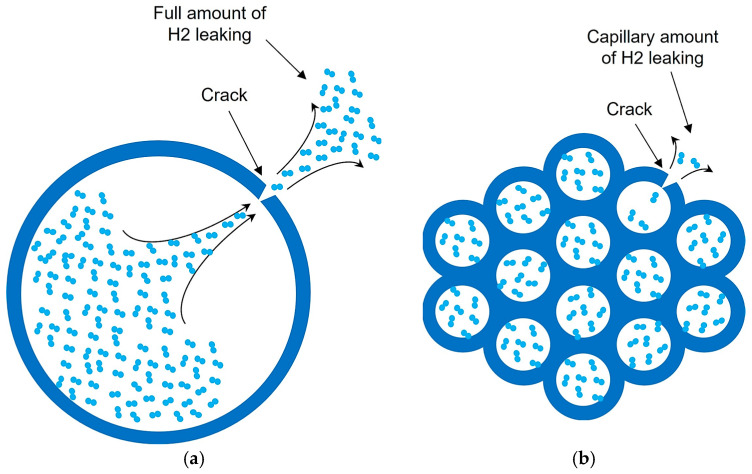
Generic representation of pressurized hydrogen release through a crack in the storage wall: (**a**) Standard hydrogen storage tank in section cut; (**b**) capillary array hydrogen storage in section cut.

Moreover, capillary array systems enhance safety. In the event of a failure, only the hydrogen within the affected capillaries is exposed to the surrounding environment, rather than the entire storage system’s contents. This contrasts sharply with standard tanks, where a rupture typically releases all stored hydrogen, posing significant safety risks (behavior presented in Figure 2). The compartmentalized design of capillary arrays mitigates this hazard, offering a safer alternative for high-pressure storage.

In addition to improved safety and efficiency, the lightweight nature of capillary arrays could lead to significant advantages in applications requiring mobility, such as automotive hydrogen storage. The reduced weight and increased storage capacity directly translate to enhanced vehicle range and lower energy consumption. Furthermore, the scalability of capillary arrays enables their adaptation to a wide variety of use cases, from compact portable storage systems to large-scale industrial applications.

The decision to focus on capillary array systems in this study is based on their potential to achieve the U.S. Department of Energy’s (DOE) ultimate targets for hydrogen storage. Capillary arrays offer a superior balance between weight, storage capacity, and cost-effectiveness compared to conventional high-pressure tank technologies.

### 2.2. Material Selection

After selecting the capillary array system as the preferred hydrogen storage technology for onboard applications, the next step is to analyze the available materials. The goal is to assess and identify those materials that offer the best combination of strength and weight.

In capillary arrays operating at high pressures (up to 700 bar), the materials must possess excellent mechanical strength to withstand internal pressure while maintaining a low density to maximize the overall energy efficiency of the storage system. This section evaluates the most common material categories for potential use in this technology, highlighting their key mechanical properties, like tensile strength and density.

Previous research [20] compares various materials based on their tensile strength (MPa) and density (g/cm^3^). This kind of analysis is crucial for evaluating the suitability of materials in engineering applications, particularly in fields like hydrogen storage, where weight-to-strength ratios are critical. The first outcome is that fiberglass offers a combination of high tensile strength, ranging from approximately 2000 MPa to 6000 MPa, and moderate density, between 2.0 g/cm^3^ and 2.6 g/cm^3^. This balance makes fiberglass highly attractive for lightweight and strong components. Its performance places it as one of the top materials for applications where strength is paramount but without significantly increasing the system’s weight. Next, Kevlar demonstrates lower tensile strength than fiberglass, at approximately 3000–3500 MPa, but compensates with a much lower density, at approximately 1.4 g/cm^3^. This makes Kevlar an excellent choice for applications where reducing weight is a primary concern while maintaining reasonable strength. On the other hand, magnesium alloys and polymers (as nonfiber shapes) show lower tensile strength values, between 100 MPa and 300 MPa, coupled with lower densities (approximately 1 g/cm^3^ to 2 g/cm^3^). Although these materials are not as strong as fiberglass or Kevlar, they are advantageous when weight reduction is a critical factor. They may not meet the demands of high-pressure hydrogen storage applications due to their limited strength, but good performances are expected from polymers if they behave like glass when reduced to fiber shape. Besides this, polymers are commonly used for type IV tanks, so they pose a high potential for further investigation. Aluminum alloys (usually used for type I to type III tanks) and titanium alloys exhibit a higher density (between 3 g/cm^3^ and 4.5 g/cm^3^) but offer substantial tensile strength in return, particularly titanium alloys, which demonstrate high strength (up to 1700 MPa). While these materials are denser, they are often used in structural applications requiring a combination of strength, durability, and resistance to deformation. This tradeoff between strength and density is common in materials used in load-bearing applications, including pressure vessels for hydrogen storage. At higher density are ceramics, which, despite their quite high density (ranging from 2.0 g/cm^3^ to 6.0 g/cm^3^), show moderate tensile strength, though they may be more brittle than other materials. Finally, high-strength and general steels (usually used for type I to type III tanks) appear in the higher density range (approximately from 7 g/cm^3^ to 8 g/cm^3^) but with excellent strength, making them suitable for heavy-duty, high-pressure applications.

This analysis clarifies that polymers and fiberglass are highly suitable for further investigation, particularly for applications requiring lightweight yet strong materials. Furthermore, it demonstrates that glass and polymers offer an optimal balance between tensile strength and low density, which is critical for onboard hydrogen storage solutions. Their properties suggest they can provide the structural integrity needed to withstand high pressures while contributing minimally to the overall system weight. Therefore, these materials stand out as the most promising candidates for improving hydrogen storage efficiency, so the next part is identifying the most suitable polymer and glass types.

#### 2.2.1. Selection of Potential Types of Polymers

Following the decision to explore polymers as primary materials for hydrogen storage in capillary arrays, the next step involves a detailed assessment of various polymer types. Density and ultimate tensile strength (UTS) are critical parameters to optimize gravimetric and volumetric hydrogen storage capacity. The materials must withstand high internal pressures while maintaining a lightweight structure, making these two properties central to our analysis.

**Figure 3:**
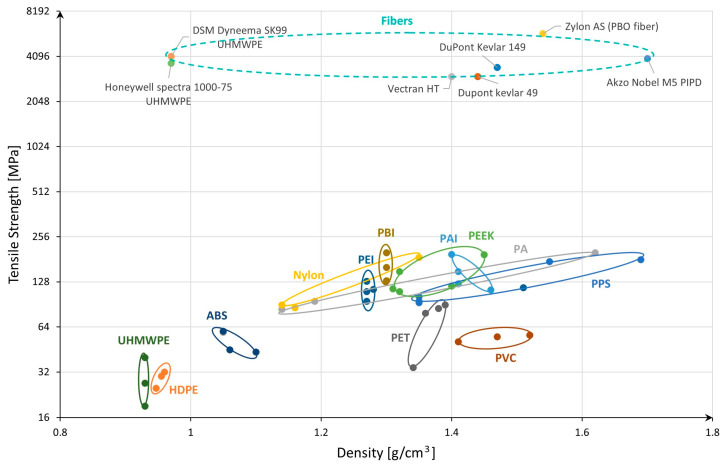
Overview of different types of polymers.

To provide a clear visualization of the performance of different polymers, we will plot the density (g/cm^3^) on the x-axis and ultimate tensile strength (UTS, MPa) on the y-axis for several commercially available polymer types, based on literature and datasheets [21,22,23,24,25], as can be seen in Figure 3. This will allow us to compare their mechanical properties and identify the most promising candidates for use in hydrogen storage capillary arrays. For better visualization, a logarithmic scale was used for the y-axis.

Figure 3 presents an overview of various polymers and their respective ultimate tensile strength to density. From the chart, it is evident that certain polymers, especially polymer fibers, stand out with significantly higher tensile strength compared to others. For example, Zylon AS (PBO fiber) and Akzo Nobel’s M5 PIPD show the highest ultimate tensile strength, exceeding 5000 MPa in the case of the first one. These materials, although dense, are incredibly strong, making them potential candidates for high-performance applications, particularly where strength is critical, such as in advanced composite materials.

Similarly, Kevlar (Dupont Kevlar 149 and Kevlar 49) and Vectran HT also demonstrate high tensile strength, though with quite similar densities. Kevlar is well-known for its applications in ballistic armor and aerospace, indicating its suitability for applications where both strength and lightweight properties are desired.

Polymers like UHMWPE (Ultra-High Molecular Weight Polyethylene) in fiber shape, used in high-strength applications like ropes and ballistic vests, offer a unique combination of relatively low density (below 1 g/cm^3^) and moderately high tensile strength (ranging approximately 4000 MPa for DSM Dyneema SK99). This makes them suitable for applications requiring lightweight but strong materials, a characteristic that is essential in hydrogen storage tanks for reducing the overall weight while maintaining structural integrity.

Lower-density polymers, such as HDPE (High-Density Polyethylene), PEI, PBI, PAI, Nylon (PA), and PEEK, balance moderate strength and lower density. These materials are versatile and can be used in a variety of engineering applications, including liners for type IV hydrogen tanks. While they do not offer the extreme strength of fiber-reinforced materials, they are still capable of providing sufficient mechanical properties for less demanding applications, or as part of composite structures.

The chart also indicates that polymers such as PVC, PET, and ABS exhibit lower tensile strength and are more suitable for nonstructural applications or applications where mechanical stress is not a primary concern. While they are not typically considered for high-strength applications, their ease of processing and affordability make them common in everyday plastic products.

From this chart, the highest-strength polymer fibers like Zylon, Kevlar, and UHMWPE are at the top in terms of tensile strength. Meanwhile, materials like Nylon, PEEK, PBI, and PEI, which offer a balance of good strength and lower density, may be more appropriate for applications like hydrogen storage liners where both weight and strength are critical. Therefore, besides the fiber types, polymers like PEEK, PBI, PAI, and even PEI could be strong candidates for further investigation when considering materials for capillary arrays in hydrogen storage solutions, based on their mechanical properties profile in the chart.

#### 2.2.2. Selection of Potential Types of Glass

This section focuses on selecting the glass types holding the greatest potential for use in capillary array hydrogen storage systems. The key properties we are evaluating are density and ultimate tensile strength, which are critical factors in ensuring the material can withstand high-pressure hydrogen storage while maintaining structural integrity and minimizing weight. By carefully considering these properties, we aim to identify glass materials that offer the best combination of strength and low density, making them ideal candidates for advanced storage solutions.

The data used in this analysis comes from an existing evaluation in the literature [20], where various glass types were compared based on their density (g/cm^3^) and ultimate tensile strength (MPa).

Different glass types show a broad range of tensile strengths, spanning from less than 1000 MPa to over 6000 MPa, and densities between 2.1 and 2.7 g/cm^3^. The most striking observation is the tensile strength of pure silica (quartz) glass fibers, reaching close to 6000 MPa, making it the strongest glass by a significant margin. Despite its high strength, its density remains approximately 2.2 g/cm^3^, indicating that it combines lightweight characteristics with exceptional strength, a critical requirement for applications such as hydrogen storage capillary arrays. S-glass fibers, with tensile strengths of approximately 4500 MPa and a density of approximately 2.5 g/cm^3^, also stand out as strong candidates for demanding applications. These fibers, often used in aerospace and defense, exhibit high tensile strength without an excessive increase in density. Similarly, while somewhat lower in tensile strength (approximately 3500 MPa and 2000 MPa, respectively), R-Glass and C-Glass fibers still offer a strong balance of strength and density, making them potential materials depending on the application’s requirements. On the lower end of the strength spectrum, soda-lime glass and borosilicate glass, commonly used for household and laboratory applications, have significantly lower tensile strengths, generally under 1000 MPa. This low strength, with their relatively higher density, makes them less suitable for high-performance structural applications like hydrogen storage.

Given the performance metrics displayed, the most promising types of glass for capillary arrays in hydrogen storage systems without reinforcement would be pure silica (quartz) glass fibers due to their exceptional tensile strength and low density. S-glass fibers are another strong candidate, balancing high tensile strength with relatively low density, making them suitable for applications where high mechanical loads are expected, but weight must be minimized. These materials can potentially meet the demanding requirements of hydrogen storage without the need for additional reinforcement.

Having conducted a thorough analysis of various polymers and glass materials, we have identified a select group of candidates that demonstrate the most promise for use in capillary arrays for hydrogen storage. The chosen materials, detailed in Table 2, include their respective density, ultimate tensile strength, and admissible stress. These parameters are critical for evaluating the mechanical performance and suitability of each material in high-pressure hydrogen applications. Furthermore, they will serve as essential inputs for the subsequent calculations of gravimetric and volumetric capacities, which are vital for determining the efficiency and effectiveness of hydrogen storage systems. By focusing on these specific materials, we aim to advance our study towards practical applications that meet the demanding requirements of hydrogen storage technologies.

### 2.3. Storage Capacity Calculation

**Figure 4:**
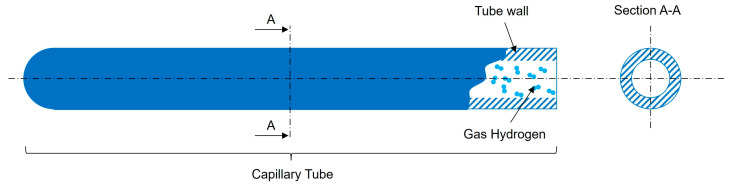
Cylindrical capillary tube in section cut.

In this part of our investigation, we will delve into the mathematical model necessary for determining the storage capacity of hydrogen using the selected materials for capillary arrays. Before proceeding with the calculations, it is crucial to establish the foundational preconditions and assumptions that will guide our analysis. These assumptions include:

1. Material Properties: The calculations will utilize the previously identified parameters for each selected material, such as density, ultimate tensile strength, and admissible stress, based on Table 2. It is assumed that these values accurately reflect the performance characteristics of the materials under the specified conditions. The values from Table 2 related to tensile strength consider the material in an ideal state without any defects, so our calculated values could be more optimistic compared to results obtained from practical experiments where material defects cannot be avoided but limited by a proper production process.
2. Safety Factors: When considering operational safety, a safety factor of 2 will be applied to account for potential variations in material performance and external stressors that may affect the integrity of the storage system.
3. Environmental Conditions: The calculations will assume standard environmental conditions for temperature and pressure, allowing for a consistent basis for comparison across different materials and configurations.
4. Geometric Configurations: To simplify the calculations, we will analyze the potential of the chosen materials through a storage system composed of a single capillary tube with a circular section cut, see Figure 4.

By establishing these preconditions, we aim to create a robust framework for our storage capacity calculations, ensuring that our findings apply to real-world hydrogen storage systems.

**Figure 5:**
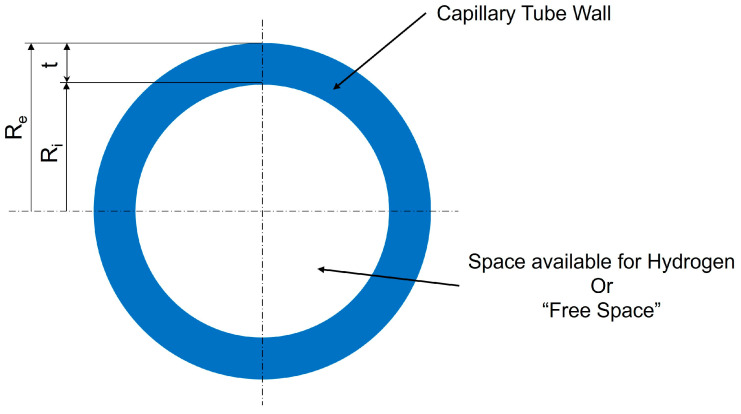
Free space representation in a capillary tube section cut.

To understand how much hydrogen a capillary array system can store, it is important to introduce the concept of “free space” within a single capillary tube. This free space represents the portion of the tube’s interior available for hydrogen storage, excluding the solid material that forms the tube walls, as can be seen in Figure 5. In simpler terms, it measures how much of the tube is hollow and capable of holding gas, compared to the total tube size.

For a cylindrical capillary tube, the amount of free space is primarily influenced by the thickness of its walls. The internal radius (R_i_) defines the hollow part, while the external radius (R_e_) includes the tube’s wall thickness. A thinner wall relative to the overall tube diameter means more space is available for storing hydrogen, making the tube more efficient for storage.

The free space can be calculated mathematically as a ratio of the internal cross-sectional area (the space where hydrogen can be stored) to the total cross-sectional area of the tube (including the material of the walls). This ratio is expressed as a percentage to make comparisons easier:

(1) $$FS=\frac{{R_{i}}^{2}}{{R_{e}}^{2}}\times 100\;[\%]$$

where R_e_ is the external radius of the capillary and Ri is the internal radius.

This percentage provides a direct indication of how much of the capillary tube’s internal volume is available for hydrogen storage. In systems designed for high storage capacity, maximizing free space is a key objective, as it directly affects the gravimetric and volumetric capacity of the storage system. However, tube wall thickness is also critical for ensuring mechanical strength, so a balance must be maintained between maximizing free space and ensuring structural integrity.

Understanding and optimizing the free space is a vital part of the design process for capillary arrays in hydrogen storage systems, as it impacts how efficiently hydrogen can be stored within a given volume.

To design and assess the structural integrity of capillary arrays used in hydrogen storage, it is essential to calculate the stress experienced by the pressure vessel walls under internal pressure. Depending on the thickness of the vessel walls relative to its internal radius, pressure vessels can be classified as having either thin walls or thick walls. The approach to calculating the stresses in these two cases differs due to the distribution of stress across the wall.

#### 2.3.1. Thin-Walled Pressure Vessels

A pressure vessel is considered “thin-walled” if its wall thickness is small compared to the internal radius (typically, if the ratio of internal radius to wall thickness is greater or equal to 10) [26]. In thin-walled vessels, stress is assumed to be uniformly distributed across the thickness of the wall, and the calculations are simplified.

$$\frac{R_{i}}{t}\geq 10$$

where t is the wall thickness.

Considering that R_e_ = R_i_ + t and R_i_ ≥10t and including Equation (1), results in pressure vessels are considered thin-walled types and free spaces higher than 83%.

For thin-walled cylindrical vessels, there are two primary types of stresses to consider:

Hoop (or circumferential) stress: This is the stress acting tangentially around the circumference of the vessel [27].

(2) $$\sigma_{h}=\frac{p_{i}\times R_{i}}{t}$$

where:

- σ_h_ is the hoop stress,
- p_i_ is the internal pressure,
- R_i_ is the internal radius of the vessel,
- t is the wall thickness.

Longitudinal stress: This is the stress acting along the length of the vessel, parallel to the vessel axis [27].

(3) $$\sigma_{l}=\frac{p_{i}\times R_{i}}{2t}$$

where:

- σ_l_ is the longitudinal stress.

Based on Equations (2) and (3), we can observe that the hoop stress is twice as large as the longitudinal stress, and it is the critical stress for evaluating the strength of thin-walled pressure vessels.

#### 2.3.2. Thick-Walled Pressure Vessels

When the wall thickness is not negligible compared to the internal radius (typically when the wall thickness is greater than 1/10 of the internal radius), the vessel is classified as “thick-walled” [26]. In thick-walled vessels, the stress distribution across the thickness is nonuniform, and a more complex analysis is required.

For thick-walled vessels, the radial and hoop stresses vary from the inner to the outer radius of the vessel. The maximum hoop stress occurs at the inner surface of the vessel and gradually decreases toward the outer surface. To calculate the stresses, Lame’s equations are used [27]:

Radial stress $\sigma_{r}$ at a distance *r* from the center of the vessel [27]:

(4) $$\sigma_{r}=\frac{p_{i}\times {R_{i}}^{2}}{{R_{e}}^{2}-{R_{i}}^{2}}\times \left(1-\frac{{R_{e}}^{2}}{r^{2}}\right)$$

At the inner surface, when r = R_i_, the radial stress becomes equal to p_i_. When r = R_e_, then the radial stress becomes 0.

Hoop stress $\sigma_{h}$ at the inner wall (maximum hoop stress) [27]:

(5) $$\sigma_{h}=\frac{p_{i}\times {R_{i}}^{2}}{{R_{e}}^{2}-{R_{i}}^{2}}\times \left(1+\frac{{R_{e}}^{2}}{r^{2}}\right)$$

At the inner surface (when r = R_i_) Equation (5) becomes:

(6) $$\sigma_{h}=\frac{p_{i}{(R_{i}}^{2}+{R_{e}}^{2})}{{R_{e}}^{2}-{R_{i}}^{2}}$$

At the outer surface (when r = R_e_) Equation (5) becomes:

(7) $$\sigma_{h}=\frac{p_{i}\times {R_{i}}^{2}}{{R_{e}}^{2}-{R_{i}}^{2}}$$

The hoop stress at the inner radius of a thick-walled pressure vessel is the critical value that must be compared with the material’s admissible stress to ensure that the vessel can safely withstand the applied pressure.

When designing a capillary tube supposed to have inner pressure, first the condition of a thin or thick wall is evaluated. If the report between internal radius and wall thickness is greater than or equal to 10, then we have a thin wall capillary, so the wall stress from Equation (2) will be compared to the admissible stress of the material from Table 2. In the case of the report between internal radius and wall thickness obtained less than 10, in this case, we have the scenario of thick wall capillary, so the wall stress from Equation (7) will be compared with the admissible stress of the material from Table 2. If the stress calculated for the analyzed dimensions of the capillary tube is less than the admissible stress of the chosen material from Table 2, then the capillary tube will not break under the inner pressure load. In the opposite case, when the calculated stress for a certain configuration of diameters and wall thickness for the capillary tube is higher than the admissible stress of the selected material, then the tube will fail under the inner pressure load.

In the end, calculating the gravimetric and volumetric capacity of a hydrogen storage system composed of capillary arrays involves determining the amount of hydrogen that can be stored relative to both the mass and the volume of the storage medium. This analysis is essential for evaluating the efficiency and practicality of using capillary arrays for hydrogen storage applications.

#### 2.3.3. Gravimetric Capacity Calculation

Gravimetric capacity refers to the mass of hydrogen that can be stored per unit mass of the storage. It is typically expressed in terms of grams of hydrogen per gram of storage material (g H₂/g storage or wt.%). The gravimetric capacity (G_capacity_) can be calculated using the following formula:

(8) $$G_{capacity}=\frac{m_{H2}}{m_{H2}+m_{storage}}\times 100\;[wt.\%]$$

where:

- m_H2_ is the mass of hydrogen stored,
- m_storage_ is the mass of the storage material.

To determine m_H2_, we can use the ideal gas law, which relates the volume of hydrogen gas at a given pressure and temperature to its mass. The ideal gas law is expressed as:

(9) PV=nRT

where:

- P is the pressure of hydrogen,
- V is the volume of hydrogen,
- n is the number of moles of hydrogen,
- R is the ideal gas constant (8.314 J/(mol·K)),
- T is the temperature in Kelvin (K).

Using the molar mass of hydrogen (M_H2_), which is approximately 2 g/mol, the mass of hydrogen can be calculated as follows:

(10) $$m_{H2}=n\times M_{H2}=\frac{PV}{RT}\times M_{H2}$$

(11) $$m_{storage}=\rho \times V_{storage}=\rho \times V_{capillary}=\rho \pi {R_{e}}^{2}L$$

where:

- ρ is the density of the material used for the storage,
- L is the length of the capillary tube.

#### 2.3.4. Volumetric Capacity Calculation

Volumetric capacity refers to the mass of hydrogen that can be stored per unit volume of the storage, typically expressed in terms of grams of hydrogen per liter of storage (g H₂/L storage). The volumetric capacity (V_capacity_) can be calculated using the following formula:

(12) $$V_{capacity}=\frac{m_{H2}}{V_{storage}}[\frac{g}{L}]$$

where:

- V_storage_ = V_capillary_ = πR_e_^2^L for a single capillary tube.

## 3. Results

### 3.1. Impact of Free Space Increase on Capillary Tube Wall Stress

**Figure 6:**
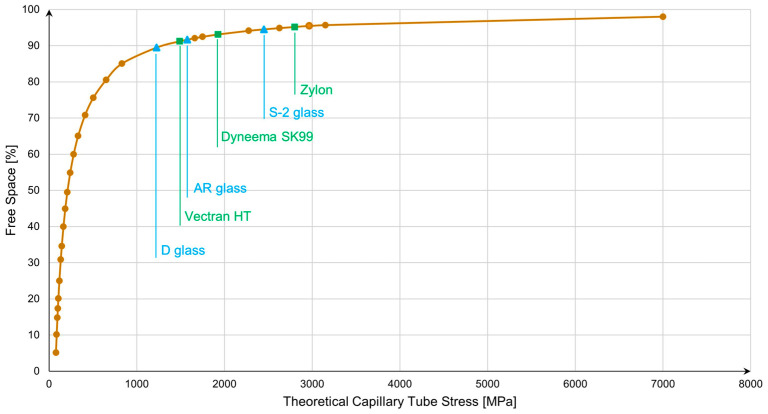
The impact of free space increases towards capillary tube stress at 700 bar storage pressure. Glass materials are represented with light blue color and polymers with green color.

The following chart from Figure 6 has been created to illustrate the relationship between the increase in free space within a capillary tube and the resulting wall stress. As the percentage of free space increases, the wall thickness decreases relative to the internal diameter, leading to a significant rise in stress for a given internal pressure. This chart highlights how hoop stress grows nonlinearly as free space increases, emphasizing the critical balance between maximizing storage capacity and ensuring structural integrity. The curve demonstrates that, beyond a certain point, further reduction in wall thickness (or increase in free space) can lead to mechanical failure, underscoring the importance of material selection and safety factor considerations in the design of capillary arrays for hydrogen storage. The critical point has been marked on the graph for each material previously selected in Table 2.

The materials investigated in this study all surpass a remarkable free space threshold of 90%, with some reaching as high as 95% (S-2 glass and Zylon AS polymer). This achievement is notable, particularly for both polymers and glass, as it demonstrates their exceptional capacity to maintain structural integrity under high internal pressure while offering an impressive amount of storage volume. Achieving such high free space percentages without compromising mechanical stability marks a significant advancement in the development of capillary arrays for hydrogen storage, highlighting the potential of these materials in next-generation energy storage systems.

### 3.2. Gravimetric Capacity Analysis

**Figure 7:**
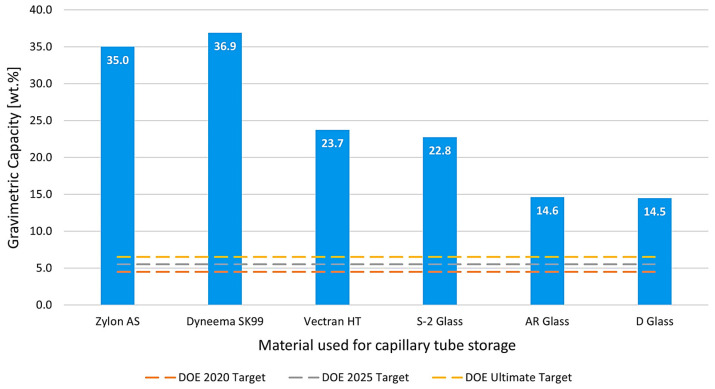
Gravimetric capacity was obtained for each type of material analyzed for the capillary tube.

The results obtained for the gravimetric capacity of the selected materials are presented graphically in Figure 7, demonstrating a comprehensive comparison against the Department of Energy (DOE) targets. Using Equation (8) and considering the critical point with maximum allowable stress and free space from Figure 6 for each material, the gravimetric capacities of the chosen polymers and glass materials were calculated, revealing their potential to significantly enhance hydrogen storage efficiency. The data indicates that these materials not only meet but also exceed the DOE’s specified targets, highlighting their suitability for advanced hydrogen storage applications.

Notably, all materials analyzed surpassed the ultimate gravimetric capacity target set by the DOE. Dyneema SK99 and Zylon excelled by an impressive factor of five to six times the target, while D and AR glass surpassed it by two times, showcasing their exceptional strength-to-weight ratios. These results underscore the significant potential of these materials in the development of high-capacity hydrogen storage systems, making them strong candidates for future applications in sustainable energy technologies.

### 3.3. Volumetric Capacity Analysis

**Figure 8:**
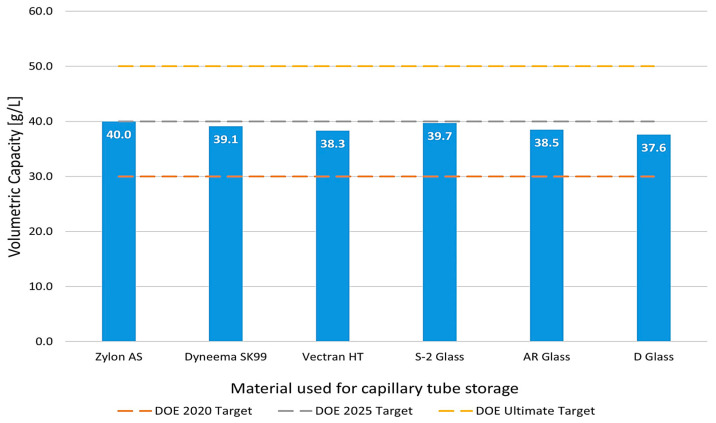
Gravimetric capacity was obtained for each type of material analyzed for the capillary tube.

The results obtained for the volumetric capacity of the selected materials are illustrated graphically in Figure 8, offering a clear comparison to the Department of Energy (DOE) targets. Utilizing Equation (12) and considering the critical point with maximum allowable stress and free space from Figure 6 for each material, the volumetric capacities of the capillary tube, composed of the chosen polymers and glass materials, were calculated, revealing their promising potential to enhance hydrogen storage systems. The calculated results demonstrate that all materials analyzed exceeded the 2020 volumetric capacity target set by the DOE, indicating significant advancements in material performance for hydrogen storage applications.

Among the materials studied, Zylon AS and S2 glass achieved the DOE’s 2025 target, reflecting their high potential for future applications. However, none of the materials reached the ultimate target outlined by the DOE. This gap suggests that further investigations, particularly the implementation of capillary arrays instead of single capillaries, could facilitate the achievement of the ultimate target. The structural design and optimization of capillary arrays may unlock enhanced volumetric capacities, paving the way for more efficient hydrogen storage solutions in next-generation energy systems.

**Figure 9:**
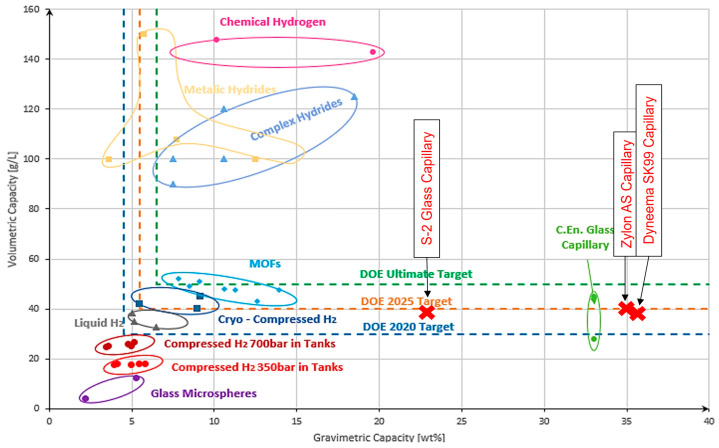
Storage capacity obtained for a single glass capillary composed of the analyzed materials compared to other existing storage technologies [17,28,29,30,31,32,33,34,35,36,37,38,39,40,41].

In the end, Figure 9 provides a comparative analysis of the gravimetric and volumetric capacities of the capillary tube composed of the top three materials examined in this study relative to existing hydrogen storage technologies. It highlights the positioning of the selected polymers and glass materials, demonstrating their superior performance in gravimetric capacity while also showing competitive volumetric capacities. This comparison underscores the potential of these materials to outperform conventional hydrogen storage methods, particularly in terms of weight efficiency, and illustrates their promising role in the evolution of advanced hydrogen storage systems.

## 4. Discussion

The main question of this study is whether the use of polymers and glass materials in capillary array systems can significantly enhance hydrogen storage capacities, both gravimetric and volumetric. The results from this study demonstrate that the selected materials, particularly high-strength polymers like Dyneema SK99 and Zylon AS, as well as specific glass types like S2 glass, show promising potential for hydrogen storage when used in capillary arrays. These materials not only meet but, in some cases, substantially exceed the DOE’s targets for gravimetric capacity and show competitive performance in terms of volumetric capacity.

Our findings suggest that the use of capillary arrays can overcome some of the limitations of conventional hydrogen storage technologies, particularly in terms of maximizing surface area and optimizing storage geometry. By doing so, the system can achieve higher hydrogen uptake while maintaining a lightweight and structurally robust design. This is a key advancement in the field, as hydrogen storage systems with higher gravimetric and volumetric efficiencies are critical for the future of sustainable energy technologies, particularly in onboard applications where weight and space are major constraints.

Our findings are supported by the mathematical model, which was employed to calculate the storage capacities based on the mechanical properties of the chosen materials. However, it is important to note that the model assumes an ideal scenario in which the materials are free from any defects. In real-life applications, material imperfections—such as microcracks or inconsistencies in manufacturing—could lead to slightly lower performance than predicted by the model. These imperfections could result in reduced tensile strength and, consequently, lower hydrogen storage capacity. Nevertheless, the impact of such defects could be mitigated through high-precision manufacturing techniques for the capillary arrays, ensuring that the materials perform closer to their theoretical potential.

In the broader context of hydrogen storage research, these findings contribute to the ongoing discussion regarding the most suitable materials for future storage systems. Previous studies have focused primarily on high-pressure tanks and materials used in type I to type IV liners, but the exploration of capillary arrays offers a new pathway to increase storage efficiency. While polymers and glass have been considered in other contexts, their potential in capillary arrays for hydrogen storage has been less explored. This study demonstrates that these materials, especially when used in novel structural configurations, can meet the increasing demands for energy storage performance.

Future research directions should focus on overcoming the current limitations in volumetric capacity by developing advanced capillary array structures that enhance storage efficiency while maintaining material strength. One promising avenue involves optimizing the geometry of capillaries, such as adjusting their size, arrangement, and wall thickness, to maximize the free space available for hydrogen storage without compromising structural integrity. This could involve exploring innovative array designs that reduce unused space and improve overall packing density.

Additionally, research should delve into the economic viability of capillary array systems compared to existing hydrogen storage solutions like type IV tanks. Economic analyses should assess factors such as material costs, manufacturing complexity, scalability, and maintenance requirements. While the theoretical capacities of polymer- and glass-based capillary arrays are impressive, translating these into cost-effective systems for mass adoption is critical for competing with current technologies.

Real-world performance testing under cyclic loading and long-term operational conditions is an essential area of investigation. Such studies would evaluate the durability of capillary array systems under repeated pressure cycles, exposure to environmental factors, and potential degradation over time. Insights from these tests could guide the selection of materials and the refinement of manufacturing processes to ensure consistent and reliable performance.

Advances in material processing techniques, such as high-precision manufacturing methods and defect minimization technologies, also hold the potential to improve system performance. For instance, additive manufacturing or advanced extrusion techniques could enhance consistency in wall thickness and material properties, reducing variability and ensuring that theoretical strengths and capacities are achieved in practical applications. In the end, this study was able to analyze and demonstrate the feasibility of the use of polymers and glass in hydrogen storage technologies composed of capillary arrays and highlights their potential to significantly improve both gravimetric and volumetric storage capacities. With further research and refinement, capillary arrays composed of these materials could become a vital component in the development of next-generation hydrogen storage systems, capable of meeting the stringent demands of future energy applications.

## 5. Conclusions

The conclusions of this study highlight the significant potential of polymers and glass materials for enhancing hydrogen storage capacities when used in capillary array systems through theoretical modeling and analysis. The results obtained between 15 and 37 wt.% (exceeding by two to five times the Ultimate DOE targets) confirm the exceptional strength-to-weight ratios of the chosen materials, making them ideal candidates for high-performance hydrogen storage systems.

In terms of volumetric capacity, Zylon AS and S2 glass were able to reach the DOE’s 2025 target, showcasing their suitability for compact storage solutions. Although none of the materials analyzed achieved the ultimate volumetric target, the results obtained between 37 and 40 g/L suggest that further optimization of capillary array configurations—rather than using single capillaries—could enhance the volumetric performance of these systems. The use of capillary arrays, which offer increased surface area and efficient geometric storage solutions, is a key factor in boosting both gravimetric and volumetric capacities.

With all analyzed materials exceeding 90% free spaces for capillary tubes, these results are a remarkable achievement for both polymers and glass. These high free space values are crucial for maximizing storage efficiency without compromising the structural integrity of the storage system.

It is important to note that the mathematical model used in this study assumes ideal material conditions, free from defects. While the theoretical results may be slightly higher than what can be achieved in real-world applications, proper manufacturing processes and quality control measures can minimize material imperfections, ensuring that the calculated capacities are attainable.

This study has shown that the selected polymers and glass materials, when integrated into capillary array systems, have the potential to revolutionize hydrogen storage by providing lightweight, strong, and highly efficient storage solutions. The gravimetric and volumetric capacities obtained in this research demonstrate the viability of these materials for next-generation hydrogen storage systems, paving the way for further developments in sustainable energy technologies.

## Figures and Tables

**Table 1:** Common hydrogen tank storage capacity versus targets overview for onboard applications.

Type	Material [17]	Gravimetric Capacity [wt.%] [18]	DOE 2020 Target [3]	DOE 2025 Target [3]	DOE Ultimate Target [3]
I	Steel/Al	1.7			
II	Steel/Al + partial composite reinforcement	2.1			
III	Steel/Al + full composite reinforcement	4.2	4.5	5.5	6.5
IV	Polymer + full composite reinforcement	5.7			

**Table 2:** Overview of the materials and properties considered for the calculations.

Material	Density [g/cm^3^]	UTS [MPa]	σ Admissible [MPa] *
Polymers			
Zylon AS (PBO fiber)	1.54	5800	2900
DSM Dyneema SK99 (UHMWPE fiber)	0.97	4100	2050
Vectran HT (LCP fiber)	1.40	3000	1500
Glass			
S-2 Glass fiber (aluminosilicate)	2.46	4890	2445
AR-Glass fiber (soda-lime)	2.70	3240	1620
D-Glass fiber (borosilicate)	2.11	2500	1250

* The admissible stress was calculated by dividing the UTS of the material by the safety factor of 2.

## Data Availability

The calculated data from this study can be provided by the corresponding author upon request.
